# Recombinant Klotho Protects Human Periodontal Ligament Stem Cells by Regulating Mitochondrial Function and the Antioxidant System during H_2_O_2_-Induced Oxidative Stress

**DOI:** 10.1155/2019/9261565

**Published:** 2019-11-28

**Authors:** Huan Chen, Xiaojun Huang, Chuanqiang Fu, Xiayi Wu, Yingying Peng, Xuefeng Lin, Yan Wang

**Affiliations:** Guanghua School of Stomatology, Hospital of Stomatology, Sun Yat-sen University, Guangdong Provincial Key Laboratory of Stomatology, Guangzhou 510055, China

## Abstract

Human periodontal ligament stem cells (hPDLSCs) are a favourable source for tissue engineering, but oxidative stress conditions during cell culture and transplantation could affect stem cell viability and stemness, leading to failed regeneration. The aim of this study was to evaluate the antioxidant and protective effects of Klotho, an antiageing protein, against cell damage and the loss of osteogenesis in hPDLSCs in H_2_O_2_-induced oxidative environments. H_2_O_2_ was used as an exogenous reactive oxygen species (ROS) to induce oxidative stress. Recombinant human Klotho protein was administered before H_2_O_2_ treatment. Multitechniques were used to assess antioxidant activity, cell damage, and osteogenic ability of hPDLSCs in oxidative stress and the effects of Klotho on hPDLSCs. Mitochondrial function was analyzed by an electron microscopy scan of cellular structure, mitochondrial DNA copy number, and cellular oxygen consumption rate (OCR). Furthermore, we explored the pathway by which Klotho may function to regulate the antioxidant system. We found that pretreatment with recombinant human Klotho protein could enhance SOD activity and reduce intracellular oxidative stress levels. Klotho reduced H_2_O_2_-induced cellular damage and eventually maintained the osteogenic differentiation potential of hPDLSCs. Notably, Klotho promoted mitochondrial function and activated antioxidants by negatively regulating the PI3K/AKT/FoxO1 pathway. The findings suggest that Klotho protein enhanced the antioxidative ability of hPDLSCs and protected stem cell viability and stemness from H_2_O_2_-induced oxidative stress by restoring mitochondrial functions and the antioxidant system.

## 1. Introduction

Periodontitis is a chronic inflammatory disease that causes the destruction of tooth-supporting tissues and the progressive loss of periodontal attachment and alveolar bone [1, 2]. Although periodontitis is the major cause of tooth loss in adults, treatments of periodontitis are far from satisfactory. Conventional infection control measures and regenerative approaches currently applied have shown limited efficacy on the restoration of periodontal supporting structures [3, 4]. In recent years, stem cell-based bioengineered therapies have been investigated as therapeutic tools in regenerative medicine [5]. Mesenchymal stem cells (MSCs) are emerging as major sources for cell-based tissue engineering due to their immunity privilege [6]. Human periodontal ligament stem cells (hPDLSCs) are MSCs from the periodontal ligament and the main candidate stem cells in periodontitis therapy. Being more accessible and possessing higher cell growth than human bone marrow stem cells (hBMMSCs) do, hPDLSCs have important homeostatic functions in vivo and display angiogenic, immunomodulatory, and multilineage differentiative capacity in vitro [7–9]. hPDLSCs have superior abilities to promote the formation of new bone, cementum, and periodontal ligament, achieving bone or periodontal tissue regeneration, as evidenced by accumulating studies [10–12]. With the osteoblastic differentiation ability, hPDLSCs are capable for repairing alveolar bone defect and periodontal intrabony defects [13, 14]. Exosomes derived from hPDLSCs also participate in mediating the immune balance and alleviating inflammatory microenvironment in periodontitis [15]. However, MSCs like hPDLSCs are placed in a harsh environment after isolation and transplantation, and the adverse microenvironment reduces their stemness and hinders their therapeutic effects [16].

Once MSCs are isolated from their original tissues or organs, they rapidly lose their vitality because of inappropriate ex vivo conditions. Additionally, long-term in vitro culture to increase cell number leads to a decreased colony-forming capacity [17, 18]. Moreover, the transplantation of MSCs decreases their survival and proliferation rates because of low oxygen and nutrient supplies [19]. In such circumstances, MSCs will produce excessive reactive oxygen species (ROS), causing DNA damage and activating the apoptosis pathway. Consequently, oxidative stress impairs the self-renewal, proliferation, and differentiation capacity of MSCs, leading to failed tissue regeneration [20]. High ROS levels can be generated by hydrogen peroxide (H_2_O_2_), hydroxyl radicals, and superoxide anions in MSCs [16, 21].

Mitochondria are generally believed to play an important role in maintaining the normal ROS level [22]. Mitochondria are not only the main sites of ROS production but also important organelles in the antioxidant system [23, 24]. Under physiological conditions, MSCs will produce basal ROS to maintain cell proliferation and differentiation, and ROS are tightly regulated by the antioxidant system [25]. Under oxidative stress conditions, excessive ROS accumulates, increasing the antioxidative needs beyond the capacity of the antioxidant defence system [26]. Additionally, excessive ROS directly damage mitochondrial structure and function, concurrently leading to cell apoptosis and death [22, 27]. Therefore, therapeutic antioxidative strategies that protect mitochondrial quality, improve antioxidative ability, and maintain the vitality and stemness of MSCs need to be developed.

Klotho is an antiageing protein that is predominantly expressed in the kidney and brain [28]. The human Klotho gene encodes three proteins: a full-length transmembrane form (mKL), a shed soluble form (sKL), and one secreted form produced by alternative splicing [29]. Klotho protein participates in the regulation of phosphate metabolism, energy metabolism, and stress resistance. Studies have found that secreted Klotho exerts antioxidative and anti-inflammatory effects and that Klotho induces the expression of the manganese superoxide dismutase (MnSOD) protein, a mitochondrial antioxidant enzyme that detoxifies superoxides [30, 31]. Sahu et al. revealed that Klotho expression is highly related to the regeneration ability of muscle progenitor cells. The knockdown of the Klotho gene reduced mitochondrial function and cellular bioenergetics, coincident with declines in tissue regeneration [32, 33]. Previous studies that reported Klotho protecting cardiac and renal cells against inflammatory responses and oxidative stress have provided new evidence for heart disease therapy and renal function recovery [30, 34, 35]. However, whether Klotho can exert a protective effect on hPDLSCs to maintain cell stemness under oxidative stress conditions is still unclear.

Therefore, the present study is aimed at investigating the effect of recombinant Klotho protein (same structure as secreted Klotho protein) on H_2_O_2_-induced oxidative stress in hPDLSCs. We demonstrated that Klotho protein enhanced antioxidative ability, reduced cellular damage, and protected osteogenic differentiation in hPDLSCs. Notably, we showed that Klotho maintained mitochondrial function and activated antioxidants, which were mediated by the AKT/FoxO1 pathway.

## 2. Materials and Methods

### 2.1. Cell Isolation and Culture

This research was approved by the Ethics Committee of Affiliated Stomatological Hospital of Sun Yat-sen University (ERC-[2016]-46). Human periodontal ligament tissues were obtained from healthy premolars from 12 donors (12-20 years old, 6 males and 6 females) with orthodontic demands. The middle section tissues of the root surfaces were scraped collected and enzymatically digested with 3 mg/mL collagenase type I and 4 mg/mL dispase for 1 h. Subsequently, the tissues were cultured in a complete *α*-minimum essential medium (Gibco, Grand Island, NY, USA) containing 10% (*v*/*v*) fetal bovine serum (Gibco), 100 U/mL penicillin, 100 *μ*g/mL streptomycin (HyClone, Logan, UT, USA), and 5 mM L-glutamine (Gibco) at 37°C in 5% CO_2_. The hPDLSCs used in this study were a mixture of cells collected from 12 donors to decrease individual variation.

### 2.2. Cell Treatment and Cell Proliferation Assay

Cells in passages 3-5 were used in the experiments. hPDLSCs were seeded in 96-well plates at the density of 3 × 10^3^ cells per well. H_2_O_2_ (Sigma-Aldrich, St. Louis, MO, USA) or Recombinant Klotho protein (PeproTech, Rocky Hill, NJ, USA) was added in the culture medium for 24 h, tested by concentration gradient. Cell viability was assessed by the methylthiazolyldiphenyl-tetrazo-lium bromide (MTT) assay. 100 *μ*L of MTT (5 mg/mL) solution was added to each well and incubated for 4 h at 37°C. Dimethyl sulfoxide was added and incubated for 10 min to dissolve the crystals. The absorbance was measured at a wavelength of 490 nm with an automatic microplate reader (BioTek, Winooski, VT, USA). To assess the effect of Klotho in reversing cell proliferation inhibition induced by H_2_O_2_, hPDLSCs were pretreated by various concentrations (0-1000 ng/mL) of Klotho for 24 hours, then treated by 100 *μ*M H_2_O_2_ for 24 hours. After treatment, cell viability was tested by MTT assay as well.

### 2.3. Intracellular ROS Production

The intracellular ROS level was measured by the ROS assay kit (Beyotime Biotechnology, China). Briefly, the hPDLSCs were collected after different treatments, then incubated with DCFH-DA (10 *μ*M/L) for 20 min at 37°C, shaking slightly every 5 min. The cells were washed with PBS, and the fluorescence was examined immediately under a fluorescence microscope (Carl Zeiss, Oberkochen, Germany) or flow cytometry (Beckman Coulter, Krefeld, Germany). Data were analyzed using CytExpert Software (Beckman Coulter).

### 2.4. Superoxide Dismutase (SOD) and Malondialdehyde (MDA) Activity Assay

The level of SOD and MDA were measured to assess the antioxidative stress effect of Klotho. The SOD activity of hPDLSCs was measured using a total SOD assay kit (Beyotime). The MDA level of hPDLSCs was tested by a lipid peroxidation MDA assay kit (Beyotime). Both were performed according to the manufacturer's instructions.

### 2.5. Cell Apoptosis Analysis

Cell apoptosis was quantitatively determined using the FITC Annexin V Apoptosis Detection Kit I (BD Biosciences, Franklin Lakes, NJ, USA) according to the manufacturer's protocol. hPDLSCs were collected and washed twice with cold PBS. Then, the cells were incubated in the solution containing FITC Annexin V, PI, and Binding Buffer for 15 min at RT (25°C) in the dark and finally analyzed by flow cytometry (Beckman Coulter).

### 2.6. Immunofluorescence

The hPDLSCs were seeded in a 12-well plate under different treatments. Cells were fixed and permeabilized using 4% paraformaldehyde and 0.1% Triton X-100. After blocking with 1% bovine serum albumin (BSA), the cells were incubated with cleaved Caspase-3 (c-Caspase-3) (Cell Signaling, USA) as primary antibody at room temperature for 4 h. Then, the cells were incubated with the secondary antibody conjugated to Alexa555 (Invitrogen, USA). Followed by incubation with Hoechst (Life Technologies, USA), the cells were imaged using fluorescence microscopy (Carl Zeiss).

### 2.7. Electron Microscopy

Mitochondrial structures were observed by transmission electron microscopy (TEM). hPDLSCs were fixed in 2.5% glutaraldehyde in 0.1 M phosphate buffer for 2 h at 4°C. Ultrathin sections were stained with uranyl acetate and lead citrate and photographed with a Tecnai G^2^ Spirit Twin TEM (FEI, USA). Sections were scanned randomly at 10 different spots per sample at ×18500 magnification. The numbers and sizes of mitochondria were measured using imaging software (ImageJ).

### 2.8. Mitochondrial Membrane Potential Detection (*ΔΨ*m)

The *ΔΨ*m of hPDLSCs was measured using a mitochondrial membrane potential assay kit with JC-1 probe (Beyotime). When live cells are incubated with JC-1, the mitochondria are driven by the *ΔΨ*m and JC-1 is rapidly taken up, raising the JC-1 concentration and the formation of aggregates (J-aggregates) within the mitochondria, which provoke red fluorescence. However, JC-1 does not accumulate in depolarized mitochondria with low *ΔΨ*m and remains in the cytoplasm as monomers, which emit green fluorescence. hPDLSCs were incubated in JC-1 working solution at 37°C for 20 min, then were washed twice with wash buffer and measured by flow cytometric analysis (Beckman Coulter) or using a fluorescent microscope (Carl Zeiss).

### 2.9. Alizarin Red Staining

hPDLSCs were seeded into 12 well plates at a density of 4 × 10^4^ cells/well. hPDLSCs were treated the same as above. After reaching 80% confluence, cells were cultured in an osteogenic induction medium (phenol red-free *α*-MEM supplemented with 10% FBS, 10 mmol/L *β*-glycerophosphate, 10 nmol/L dexamethasone, and 50 *μ*g/mL ascorbic acid) for osteogenic differentiation. The medium was replaced every other day. 14 days later, cells were stained by the Alizarin red (pH 4.2, Alfa Assar). Calcium nodules were observed via a microscope and imaged with camera. For quantitative analysis, stained nodules were dissolved with 10% (*w*/*v*) cetylpyridinium chloride (Sigma-Aldrich) and detected by an automatic microplate reader at 562 nm.

### 2.10. Alkaline Phosphatase (ALP) Activity

After 7 days of osteogenic induction, hPDLSCs were fixed and stained according to the manufacturer's instructions of the BCIP/NBT alkaline phosphatase colour development kit (Beyotime) and the images were captured with a camera. ALP activity of hPDLSCs was measured following the manufacturer's instructions using the Alkaline Phosphatase Assay kit (Beyotime). Absorbance was evaluated spectrophotometrically at 405 nm.

### 2.11. Quantitative Real-Time Reverse Transcription Polymerase Chain Reaction (qRT-PCR)

Total RNA samples were isolated from cells by using the Ultrapure RNA kit (CWBIO, China). mRNA was reverse transcribed into cDNA by a Reverse Transcriptase M-MLV Kit (TaKaRa, Japan) according to the manufacturer's instructions. RT-PCR was performed on the LightCycler® 480 Real-Time PCR System with LightCycler® 480 SYBR-Green I Master (Roche Diagnostics, Swiss). The amplification conditions were set as follows: 95°C for 10 min, 40 cycles of denaturation at 95°C for 15 s, annealing at 60°C for 20 s, and final extension at 72°C for 20 s. GAPDH was used as the internal reference gene. Gene expression was calculated using the 2^−*ΔΔ*Ct^ method. Primer sequences are listed in Table 1.

### 2.12. Western Blot Analysis

hPDLSCs were lysed in RIPA buffer at 4°C for 30 min. Protein concentrations were measured by a Pierce BCA Protein Assay Kit (Thermo Scientific; Pierce, Germany). The sample protein was separated on 10% sodium dodecyl sulfate polyacrylamide gels and electrotransferred to nitrocellulose membranes (Millipore, Germany). Subsequently, membranes were incubated in 5% nonfat milk for 1 h at room temperature and then incubated overnight with the primary antibodies at 4°C. Finally, the membranes were washed with TBST buffer and incubated with HRP-linked antibody for 1 h at room temperature, followed by detection using the GeneGnome XRQ chemiluminescence imaging system. Primary antibodies against the following were used in this study: RUNX2 (ABclonal, China), BSP (Boster, China), AKT (Cell Signaling), phospho-AKT (P-AKT) (Cell Signaling), FoxO1 (Cell Signaling), phospho-FoxO1 (P-FoxO1) (Cell Signaling), Catalase (Cell Signaling), MnSOD (Cell Signaling), and *β*-actin (Sigma-Aldrich). Relative density of protein band was analyzed by ImageJ software and normalized to *β*-actin.

### 2.13. Mitochondrial DNA (mtDNA) Copy Number

Nuclear and mitochondrial DNA of hPDLSCs was isolated according to a HiPure Tissue DNA Mini Kit using the manufacturer's protocol (Magen, China). The relative mtDNA copy number was quantified by real-time polymerase chain reaction (PCR) and normalized by simultaneous measurement of the nuclear DNA according to the method described in other studies [36]. The primer sequences L394 and H475 were used for measuring the mtDNA content, and the primers HBG1F and HBG1R were used for amplification of the single-copy nuclear *β*-globin gene (seen in Table 1). Nuclear *β*-globin was used as the internal control.

### 2.14. Mitochondrial Bioenergetic Assessment

The cellular oxygen consumption rate (OCR) was assessed in real-time using a Seahorse XFe96 Extracellular Flux Analyzer (Billerica, MA, USA). Before running the procedure, the cell medium was changed to a running medium (DMEM supplemented with 5.5 mM glucose, 1 mM sodium pyruvate, 4 mM L-glutamine, pH 7.4) and incubated at 37°C in a non-CO_2_ incubator for 1 h. The basal OCR was measured by averaging the OCR values before treating the cells with oligomycin. Total reserve capacity was calculated by the differences of OCR between treatment with FCCP and 2DG and basal values.

### 2.15. Statistical Analysis

All experiments were performed three times separately, and all data were expressed as mean ± S.D. Data were analyzed by using one-way ANOVA and SPSS20.0 software package (SPSS Inc., Chicago, IL, USA). *P* < 0.05 indicated significance.

## 3. Results

### 3.1. Recombinant Klotho Protein Protected hPDLSC Proliferation from H_2_O_2_-Induced Cytotoxicity

**Figure 1:**
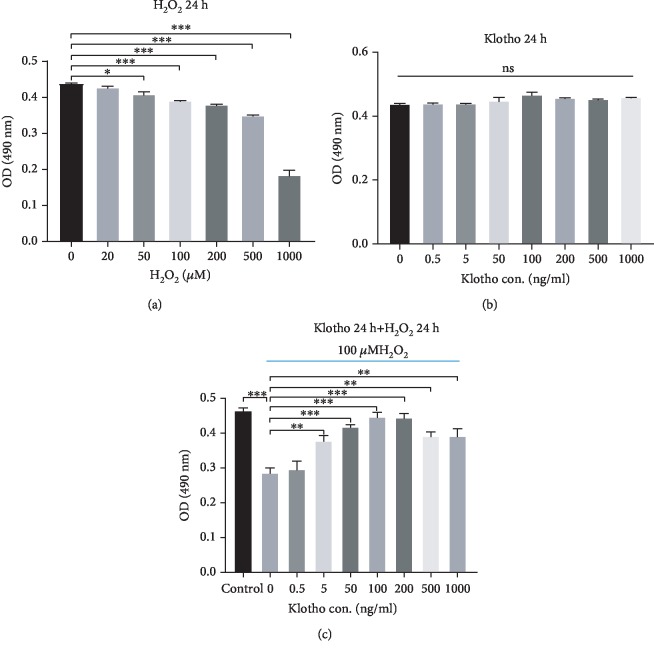
Effects of H_2_O_2_ and Klotho on hPDLSC proliferation. (a) Effect of H_2_O_2_ on the proliferation of hPDLSCs. (b) Effect of Klotho on the proliferation of hPDLSCs under normal culture conditions. (c) Effect of Klotho on the proliferation of hPDLSCs under oxidative stress conditions. Control: without Klotho or H_2_O_2_ treatment. ^∗^*P* < 0.05, ^∗∗^*P* < 0.01, and ^∗∗∗^*P* < 0.001; ns: no statistical significance.

The isolated cells were plastic-adherent and exhibited colony-forming ability. Flow cytometry analysis showed that the cells expressed CD90 and CD105 (more than 99%) and did not express CD34, CD45, and HLA-DR surface molecules (less than 1%). Moreover, the cells possessed the ability to differentiate into osteoblasts and adipocytes. These results suggested that the cells we isolated from human periodontal ligament tissue were MSCs ([Supplementary-material supplementary-material-1]). To determine the appropriate concentrations of H_2_O_2_ and Klotho, we used the MTT assay to assess the viability of hPDLSCs treated with different concentrations of H_2_O_2_ and Klotho, respectively. As shown in Figure 1(a), the proliferative activity of hPDLSCs was significantly decreased following treatment with concentrations of H_2_O_2_ at 100 *μ*M and above and was sharply reduced when the cells were exposed to 500 and 1000 *μ*M H_2_O_2_. Therefore, 100 *μ*M was chosen as the moderate H_2_O_2_ concentration to establish the oxidative stress model. The viability of hPDLSCs treated with different concentrations of Klotho was not significantly changed, suggesting that Klotho has no cytotoxicity on hPDLSCs (Figure 1(b)). To investigate whether Klotho ameliorates H_2_O_2_-induced cytotoxicity, we assessed the cell viability of hPDLSCs pretreated with different concentrations of Klotho before H_2_O_2_. The results showed that Klotho ameliorated H_2_O_2_-induced cytotoxicity in hPDLSCs, and the effect peaked at a concentration of 100 ng/mL (Figure 1(c)). Based on these results, 100 ng/mL Klotho was chosen for the subsequent experiments.

### 3.2. Recombinant Klotho Protein Attenuated Intracellular Oxidative Stress Status in hPDLSCs

**Figure 2:**
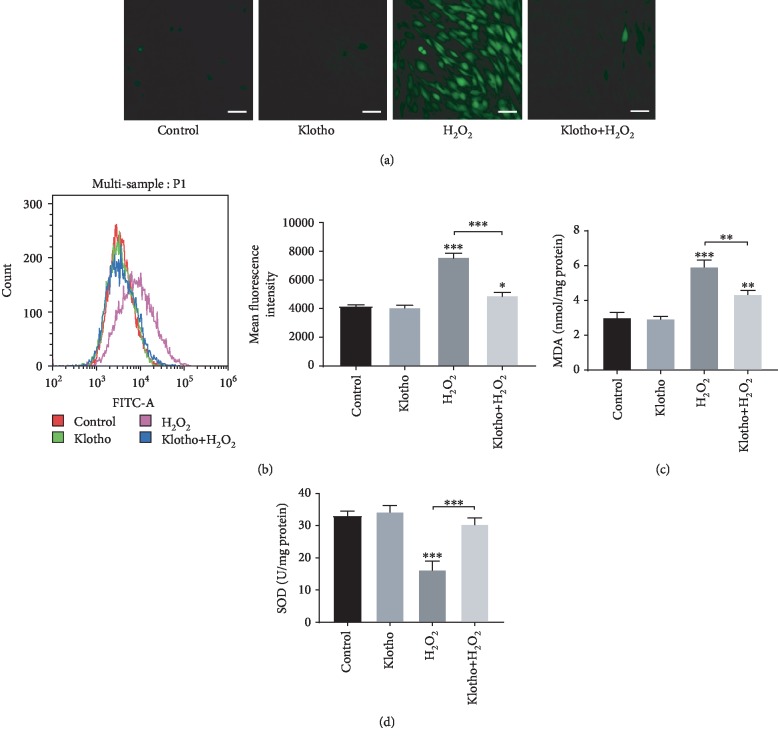
Klotho attenuated H_2_O_2_-induced intracellular oxidative stress status. SOD activity, MDA formation, and ROS production were assessed. (a, b) The level of ROS in hPDLSCs was measured by DCFH-DA through a fluorescence microscope (a) and flow cytometry (b); (c) the MDA level and (d) SOD level in hPDLSCs. Scale bars: 50 *μ*m. Magnification: 200x. ^∗^*P* < 0.05, ^∗∗^*P* < 0.01, and ^∗∗∗^*P* < 0.001 versus control group.

To verify the effect of Klotho on H_2_O_2_-induced intracellular oxidative stress status, we assessed ROS production, MDA formation, and SOD activity in hPDLSCs. ROS and MDA levels were increased, and SOD activity was impaired by H_2_O_2_ treatment compared with the control condition. However, ROS production and MDA formation were reduced by 100 ng/mL Klotho pretreatment (Figures 2(a)–2(c)), and SOD activity was also restored with the addition of Klotho (Figure 2(d)), indicating that Klotho attenuates H_2_O_2_-induced oxidative stress and exerts an antioxidative effect on hPDLSCs under mimicking oxidative stress status.

### 3.3. Recombinant Klotho Protein Reduced H_2_O_2_-Stimulated Oxidative Cellular Damages in hPDLSCs

**Figure 3:**
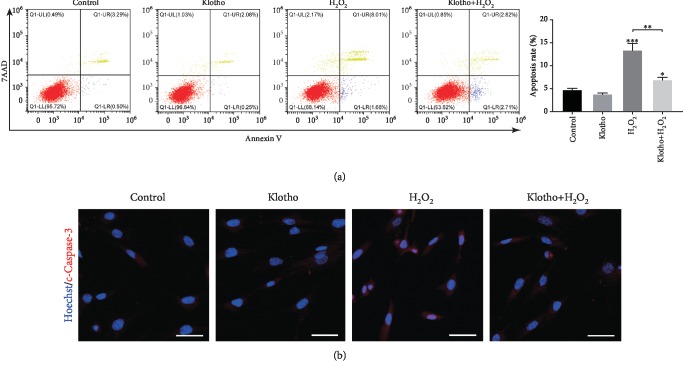
Klotho administration ameliorated H_2_O_2_-induced apoptosis in hPDLSCs. (a) Flow cytometric analysis of groups with different treatments and the percentage of Annexin V/7-AAD positive cells. (b) The expression of c-Caspase-3 in hPDLSCs with different treatments was detected by immunofluorescence staining. Scale bars: 50 *μ*m. Magnification: 400x. ^∗^*P* < 0.05, ^∗∗^*P* < 0.01, and ^∗∗∗^*P* < 0.001.

To investigate whether Klotho decreases cell apoptosis, flow cytometric analysis was performed by Annexin V staining. The results showed that Annexin V/7-AAD-positive hPDLSCs were increased in the presence of H_2_O_2_ and were significantly reduced by Klotho pretreatment (Figure 3(a)). The expression of c-Caspase-3, a proapoptotic marker, was detected by immunofluorescence staining. We found that c-Caspase-3 was activated in hPDLSCs in the presence of H_2_O_2_ and that Klotho pretreatment reduced the expression of c-Caspase-3 (Figure 3(b)).

**Figure 4:**
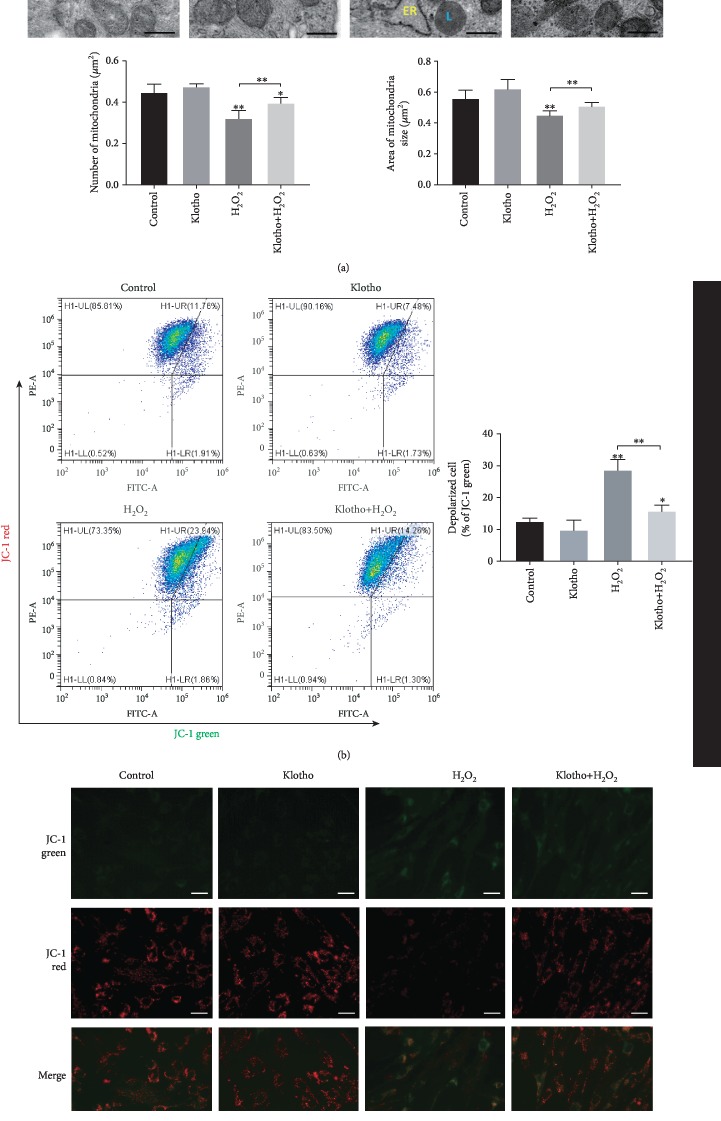
Klotho administration ameliorated H_2_O_2_-induced mitochondrial destruction in hPDLSCs. (a) Representative transmission electron microscopy images of mitochondria and their quantification in each group. (b) Flow cytometry and (c) fluorescent microscopy images of the mitochondrial membrane potential in PDLSCs with different treatments. JC-1 aggregates show red fluorescence, indicating high mitochondrial membrane potential, and JC-1 monomers show green fluorescence. M: mitochondria; L: lipid droplet; ER: endoplasmic reticulum. Scale bars: 0.5 *μ*m (a) or 30 *μ*m (c). Magnification: 18500x (a) or 200x (c). ^∗^*P* < 0.05, ^∗∗^*P* < 0.01, and ^∗∗∗^*P* < 0.001.

To identify the effect of Klotho on mitochondrial structure, we studied morphological changes in the mitochondria of hPDLSCs by electron microscopy. Ultrastructural analysis revealed that H_2_O_2_ treatment caused a reduction in the number, and size of mitochondria, and Klotho pretreatment overcame those changes (Figure 4(a)). The collapse of mitochondrial membrane potential is often observed during early apoptosis [37]. Next, the *ΔΨ*m of hPDLSCs administered different treatments was measured (Figure 4(b)). The results showed that the number of depolarized cells with low *ΔΨ*m was increased in the H_2_O_2_ group, but Klotho administration ameliorated this impairment, indicating that Klotho may exert an effect on mitochondrial structure maintenance.

### 3.4. Recombinant Klotho Protein Alleviated the Suppression of H_2_O_2_-Induced Osteogenic Differentiation in hPDLSCs

**Figure 5:**
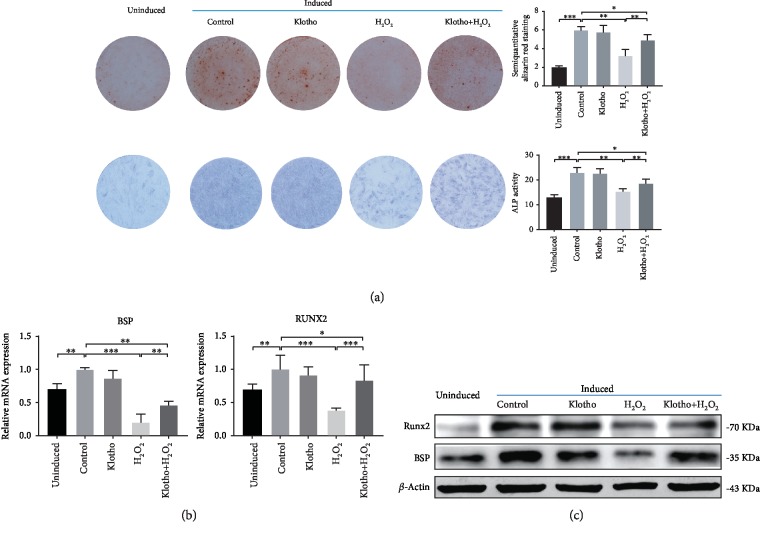
The effect of Klotho on the osteogenic differentiation of hPDLSCs stimulated by H_2_O_2_. (a) Alizarin red staining and ALP activity assay. (b) qRT-PCR analysis of RUNX2 and BSP mRNA expression. (c) Western blot analysis of RUNX2 and BSP gene expression. ^∗^*P* < 0.05, ^∗∗^*P* < 0.01, and ^∗∗∗^*P* < 0.001.

To verify the effect of Klotho on the osteogenic differentiation of hPDLSCs in H_2_O_2_-induced oxidative stress, we assessed extracellular matrix (ECM) mineralization, ALP activity, and the mRNA and protein expression of osteogenic-related markers. The formation of mineral nodules and ALP activity was inhibited under H_2_O_2_ stimulation and was reversed by Klotho pretreatment (Figure 5(a)). RUNX2 and BSP play crucial roles in osteogenic differentiation. RT-PCR and Western blot analysis showed that the expression of RUNX2 and BSP was downregulated remarkably in the H_2_O_2_ group, and Klotho pretreatment elevated osteogenic gene expression (Figures 5(b) and 5(c)). These results demonstrated that Klotho protected osteogenic potential, which represents the stemness of hPDLSCs, from H_2_O_2_-induced oxidative stress.

### 3.5. Recombinant Klotho Protein Contributed to the Regulation of Mitochondrial Dysfunction in hPDLSCs

**Figure 6:**
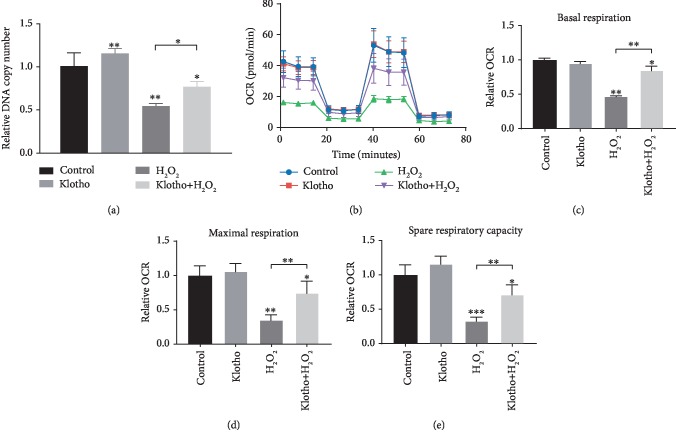
The effect of Klotho on the mitochondrial function of hPDLSCs stimulated by H_2_O_2_. (a) mtDNA copy number analysis by qRT-PCR. (b) Seahorse analysis of hPDLSCs. The areas under the curve for respiration (OCR) are shown, and (c–e) the basal, maximal, and spare respiration capacity were calculated from the OCR. ^∗^*P* < 0.05, ^∗∗^*P* < 0.01, and ^∗∗∗^*P* < 0.001.

We have demonstrated that Klotho exerts protective effects on hPDLSC mitochondrial structure. Next, we evaluated whether Klotho protein has a direct influence on hPDLSC mitochondrial function in an oxidative environment. qRT-PCR results showed that mitochondrial DNA (mtDNA) copy number, a marker representing mitochondrial viability, was significantly reduced in the 100 *μ*M H_2_O_2_ group compared with the control group and could be ameliorated by Klotho pretreatment (Figure 6(a)). The OCR of cells was assessed and shown in Figures 6(b)–6(e). The OCR of Klotho-pretreated cells was higher than that of cells treated with H_2_O_2_ alone. Klotho-pretreated hPDLSCs showed significantly higher basal respiration, maximal respiration, and spare respiratory capacity. Overall, these data supported the hypothesis that Klotho could protect hPDLSC mitochondrial bioenergetics from H_2_O_2_-induced oxidative stress.

### 3.6. Recombinant Klotho Protein Enhanced the Antioxidant System by Regulating the PI3K/AKT/FoxO1 Pathway

**Figure 7:**
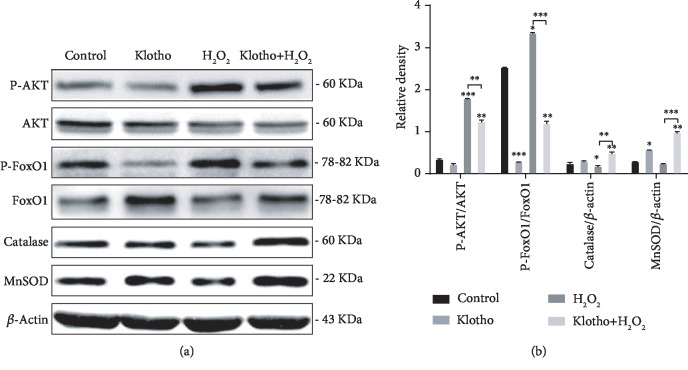
Klotho enhanced the Catalase and MnSOD expression by regulating the AKT/FoxO1 pathway in H_2_O_2_-treated hPDLSCs. Western blotting results (a) and relative density (b) of the protein expression of AKT, phosphorylated AKT (p-AKT), phosphorylated FoxO1 (p-FoxO1), FoxO1, Catalase, and MnSOD. ^∗^*P* < 0.05, ^∗∗^*P* < 0.01, and ^∗∗∗^*P* < 0.001.

The PI3K/AKT signalling pathway plays a crucial role in stem cell self-renewal and differentiation, and its downstream target FoxO1 is tightly related to antioxidant genes, such as MnSOD and Catalase [38, 39]. Therefore, we explored the effect of Klotho on PI3K/AKT/FoxO1 pathway regulation. Western blot analysis showed that H_2_O_2_ activated the AKT/FoxO1 pathway in hPDLSCs, promoting the phosphorylation of AKT and FoxO1. Additionally, H_2_O_2_ reduced total FoxO1 expression, leading to the reduced expression of Catalase and MnSOD. Notably, the H_2_O_2_-induced decrease in enzymatic antioxidants was reversed by Klotho pretreatment, which is consistent with the elevated FoxO1 expression (Figures 7(a) and 7(b)). Taken together, the results show that Klotho negatively regulates the PI3K/AKT/FoxO1 signalling pathway and subsequently enhances FoxO1-mediated antioxidant expression.

## 4. Discussion

Since the concept of tissue engineering was proposed two decades ago [40], MSCs have been extensively studied because of their significant potential in the field of regenerative medicine [41]. However, so far, MSCs are much farther from reaching clinical utility in regenerative medicine than they are in immunomodulation [42]. One of the important reasons is the effect of oxidative stress on MSC ex vivo expansion and in vivo engraftment, leading to the loss of stemness and low survival rate in transplant sites, eventually causing failed tissue regeneration [16, 21]. Excessive ROS in MSCs is the typical sign for oxidative stress in the injured microenvironment.

In our study, we aimed to find a solution to improve hPDLSC bioactivity under oxidative stress. Accumulating evidence suggests that excessive oxidative stress and diminished antioxidant defences could contribute to age-related tissue damage and diseases [43]. The antiageing protein Klotho exerts antioxidative effects in cells [31]. In the present study, we investigated whether exogenous treatment with Klotho protein could protect against H_2_O_2_-induced oxidative injury in hPDLSCs.

H_2_O_2_ has been extensively used on in vitro models to induce oxidative stress [44]. ROS overproduction and failed antioxidant defence lead to redox imbalance and increased oxidative stress [45]. H_2_O_2_ causes cell oxidative damage mainly by causing excessive ROS and attacking the antioxidant system [46]. We found that 100 ng/mL Klotho protein showed the greatest protection against oxidative stress. The excess accumulation of ROS damages polyunsaturated fatty acids and leads to higher levels of MDA, which is a major end product of membrane lipid peroxidation [47]. Lipid peroxidation may cause cell membrane damage, resulting in a decreased membrane permeability and fluidity and eventual cell death [48]. Exogenous proteins exert antioxidative effects by promoting the expression of antioxidant enzymes such as SOD [49]. By detecting the level of oxidative stress biomarkers [50], we found that Klotho alleviated oxidative stress in hPDLSCs by reducing ROS production and lipid oxidative damage, while the activities of antioxidant enzymes such as SOD were maintained (Figure 2).

The TEM observations showed that the hPDLSCs pretreated with Klotho maintained mitochondrial ultrastructure (Figure 4(a)). Klotho reduced vacuolated mitochondria and compromised cristae structure under H_2_O_2_-induced oxidative stress. The mitochondrial membrane depolarization induced by H_2_O_2_ treatment was also recovered by Klotho treatment (Figures 4(b) and 4(c)). A reduction in mitochondrial membrane potential induces the opening of the mitochondrial permeability transition pore and the release of proapoptosis factors, such as cytochrome c, into the cytosol [51]. These previous results explained the findings in our study that cellular apoptosis paralleled mitochondrial damage, and Klotho reduced the H_2_O_2_-induced cell apoptosis rate and caspase 3 expression level (Figure 3). H_2_O_2_ exposure can induce mitochondrial fission and apoptosis by converting cytochrome c into an oxidant-generating peroxidase, which eventually impairs cellular dynamics [52]. The present results show that the Klotho protein attenuated the hPDLSC cellular damage and cell apoptosis induced by oxidative stress by protecting mitochondrial structure. Other studies showed a similar function of Klotho, reporting that it reduces cell apoptosis by decreasing mitochondrial ROS production [53, 54].

hPDLSCs possess predominant osteogenic differentiation potentials and are supposed to be ideal cell sources for periodontal regeneration [55]. However, H_2_O_2_ impairs osteogenic capacity by suppressing ALP activity, mineralization, and osteogenic gene levels, including the levels of RUNX2, BSP, and OCN [56]. A previous report showed that the addition of antioxidants reduces ROS generation and eliminates oxidative stress, strikingly elevating the expression of RUNX2, ALP, and OPG [57]. We found that Klotho increased the osteogenic differentiation of PDLSCs under oxidative stress, promoting mineralized nodule deposition, ALP activity, and RUNX2 and BSP expression (Figure 5). From the data, we concluded that Klotho protected the osteogenic differentiation of hPDLSCs against oxidative stress, showing the potential to solve problems in periodontal regeneration.

However, it is not known how Klotho exerts these effects on hPDLSC osteogenesis. Studies have found that increased mtDNA copy number and enhanced mitochondrial respiration were observed in differentiated MSCs, indicating that mitochondria may participate in the osteogenic differentiation of MSCs [58]. We further investigated mitochondrial function in different groups, and the results demonstrate that the antioxidative effect promoted by Klotho coincides with the regulation of mitochondrial bioenergetics. Mitochondrial function, as assessed by mtDNA damage and OCR, showed that Klotho-treated cells had less mtDNA loss and significantly higher maximal respiration and spare respiratory capacity than did control cells (Figure 6). Spare capacity represents reserve bioenergetic capacity and is calculated as the difference between the basal and maximal OCR, indicating the ability of a cell to respond to stress [59]. In general, mitochondrial quality is controlled by interconnected systems, including elements for fighting oxygen-mediated toxicity, maintaining mitochondrial proteostasis, and regulating mitochondrial morphology, location, and number [23]. Taken together, these data support the hypothesis that Klotho administration protected the quality and bioactivity of mitochondria in hPDLSCs, and these effects may reduce H_2_O_2_-induced oxidative damage.

Next, we examined whether the Klotho-induced effects were mediated through the PI3K/AKT/FoxO1 pathway. The PI3K/AKT pathway has been reported to play an important role in the biological behaviour of MSCs [60]. The present study verified that Klotho increased the expression of Catalase and MnSOD. Klotho negatively mediated the PI3K/AKT pathway to regulate FoxO1-mediated enzymatic antioxidant expression (Figure 7). The PI3K/AKT signalling pathway regulates FoxO through phosphorylation [29, 61, 62]. The AKT-mediated phosphorylation of FoxO1 inhibits FoxO1 activity by promoting its nuclear exportation and proteasomal degradation [63]. FoxO1 can upregulate MnSOD and Catalase expression. However, it remains unclear whether the ability of Klotho to protect mitochondria and to confer oxidative stress resistance is entirely dependent on Catalase and MnSOD induction. Further investigation, such as gene overexpression and knockdown of Klotho, should be performed to verify this hypothesis and molecular mechanism.

## 5. Conclusion

Thus far, the findings imply the protective effects of recombinant Klotho protein on hPDLSCs under oxidative conditions. Pretreatment with Klotho attenuated H_2_O_2_-induced cell damage and decreased osteogenesis by restoring the mitochondrial antioxidant system through the PI3K/AKT/FoxO1 pathway. Our study indicated that Klotho could be a promising option for protecting hPDLSCs against oxidative injuries and ensuring cell stemness for osteogenesis and tissue regeneration engineering.

## Figures and Tables

**Table 1:** Primer sequences used in quantitative real-time reverse transcription polymerase chain reaction.

Gene target	Sequence
RUNX2	Forward: 5′-TGGTTACTGTCATGGCGGGTA-3′
	Reverse: 5′-TCTCAGATCGTTGAACCTTGCTA-3′

BSP	Forward: 5′-GAACCACTTCCCCACCTTTTG-3′
	Reverse: 5′-ATTCTGACCATCATAGCCATCG-3′

GAPDH	Forward: 5′-GGAGCGAGATCCCTCCAAAAT-3′
	Reverse: 5′-GGCTGTTGTCATACTTCTCATGG-3′

mtDNA	Forward: 5′-CACCAGCCTAACCAGATTTC-3′
	Reverse: 5′-GGGTTGTATTGATGAGATTAGT-3′

Nuclear *β*-globin	Forward: 5′-GCTTCTGACACAACTGTGTTCACTAGC-3′
	Reverse: 5′-CACCAACTTCATCCACGTTCACC-3′

## Data Availability

The data used to support the findings of this study are included within the article.

## Supplementary Materials

Supplementary Materials Figure S1: characterization of hPDLSCs.
