# Supplementary material for: Recombinant Klotho Protects Human Periodontal Ligament Stem Cells by Regulating Mitochondrial Function and the Antioxidant System during H2O2-Induced Oxidative Stress
Source: Oxid Med Cell Longev. 2019 Nov 28;2019:9261565. doi: 10.1155/2019/9261565 (PMC6914990; doi:10.1155/2019/9261565)
Supplement: Supplementary Materials — Figure S1: characterization of hPDLSCs. [file 9261565.f1.pdf]

Supplementary Materials

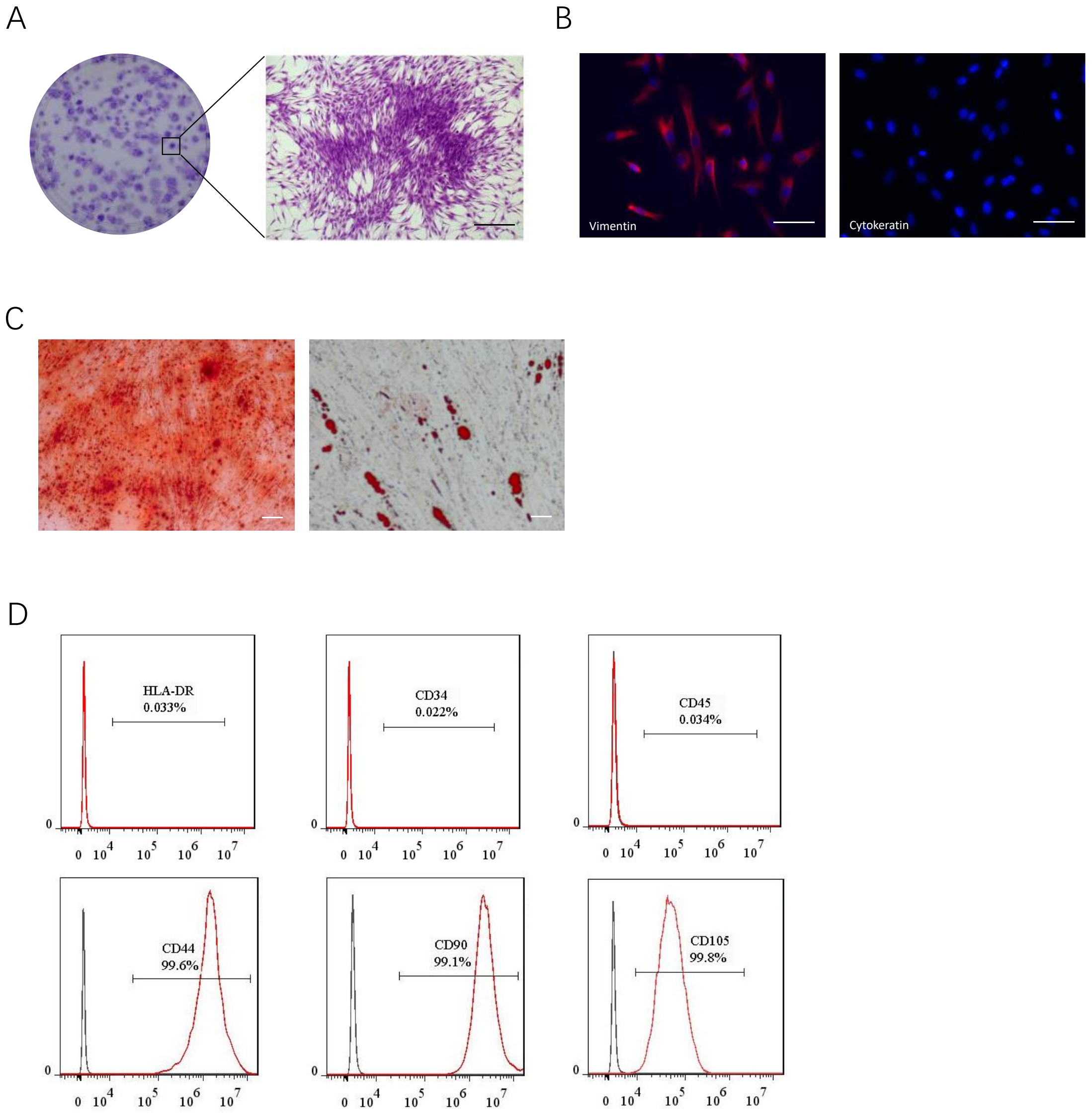

Marker	HLA-DR	CD34	CD45	CD44	CD90	CD105
Percentage	0.033%	0.022%	0.034%	99.6%	99.1%	99.8%

Figure S1: Characterization of hPDLSCs. (A) Representative images of a single colony-forming unit of hPDLSCs at 12 days. (B) Immunofluorescence staining demonstrated that hPDLSCs positively expressed vimentin and did not express cytokeratin. (C) The ability of hPDLSCs to differentiate into multiple cell types, as demonstrated by Alizarin red and Oil red O staining under specific differentiation conditions for osteoblasts or adipocytes. (D) Cell surface markers of hPDLSCs were detected by flow cytometry. Scale bars: 300 μm (A) or 50 μm (B, C).
